# Supplementary material for: Facilitating analysis and dissemination of proteomics data through metadata integration in MaxQuant
Source: Nat Commun. 2025 Sep 25;16:8421. doi: 10.1038/s41467-025-64089-4 (PMC12462489; doi:10.1038/s41467-025-64089-4)
Supplement: Supplementary file 1 — Supplementary Information [file 41467_2025_64089_MOESM1_ESM.pdf]

Supplementary Methods

Step-by-step protocol for exporting the SDRF file in MaxQuant and using SDRF to annotate output tables in Perseus

These features have been implemented since MaxQuant v2.7.0 and Perseus v2.1.5. The video tutorial can be viewed on YouTube at https://youtu.be/fHCPOBXXRp8.

1. Set up the MaxQuant run

1.1 Load the raw files and set experiments (also set fractions if you have them)

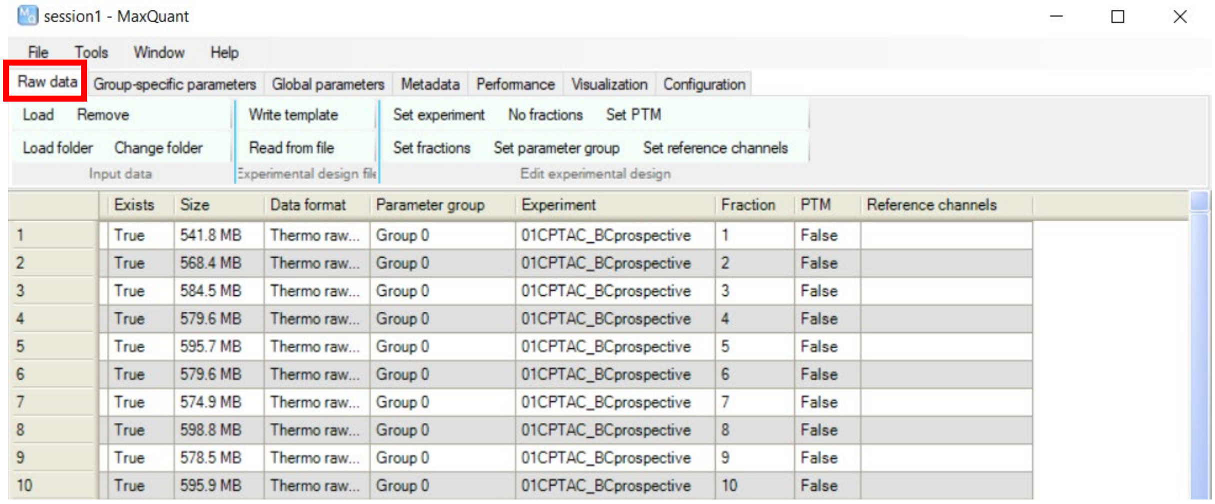

1.2 Set the Group-specific parameters for the run

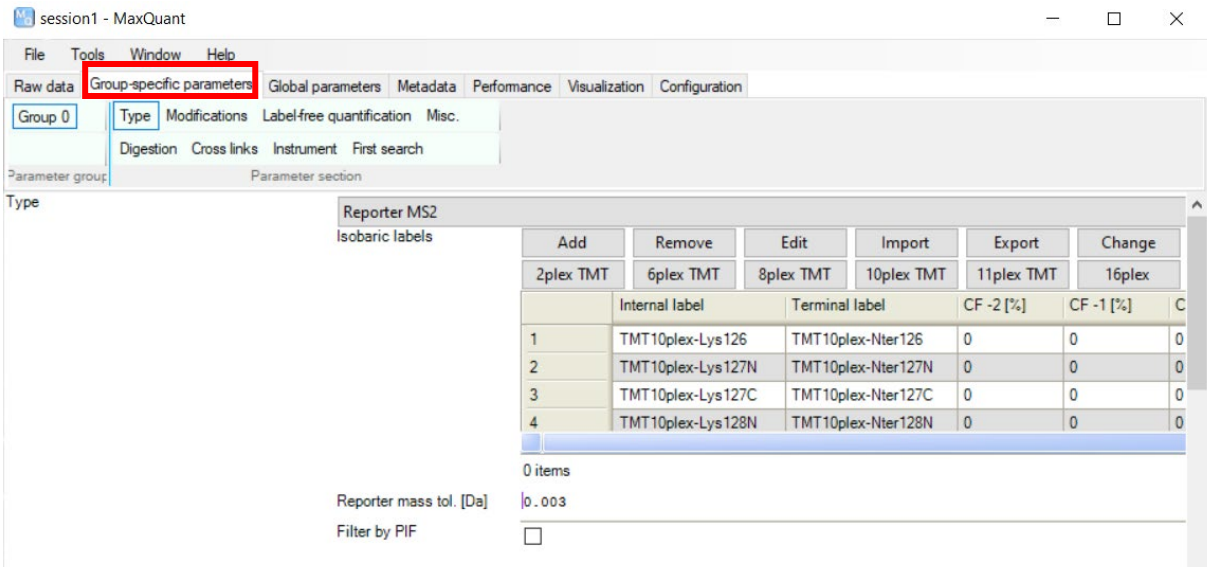

1.3 Set the Global parameters for the run

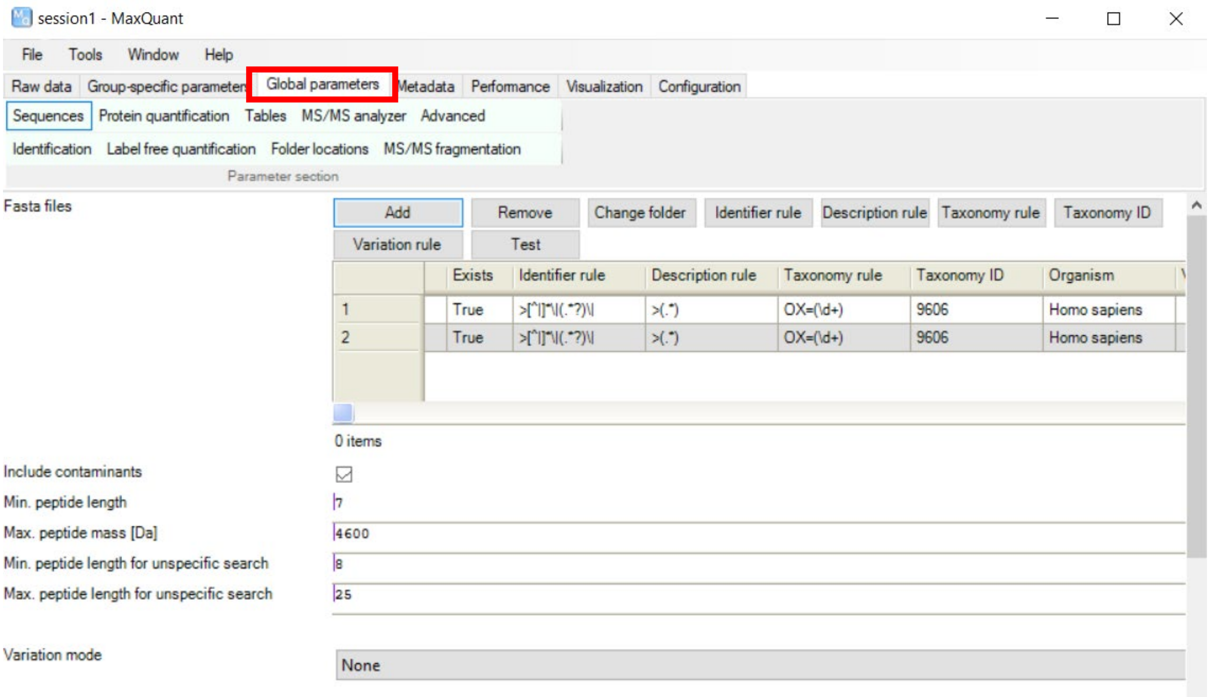

2. Export SDRF in MaxQuant

2.1 Create the metadata table by clicking "Refresh" under the "Metadata" tab

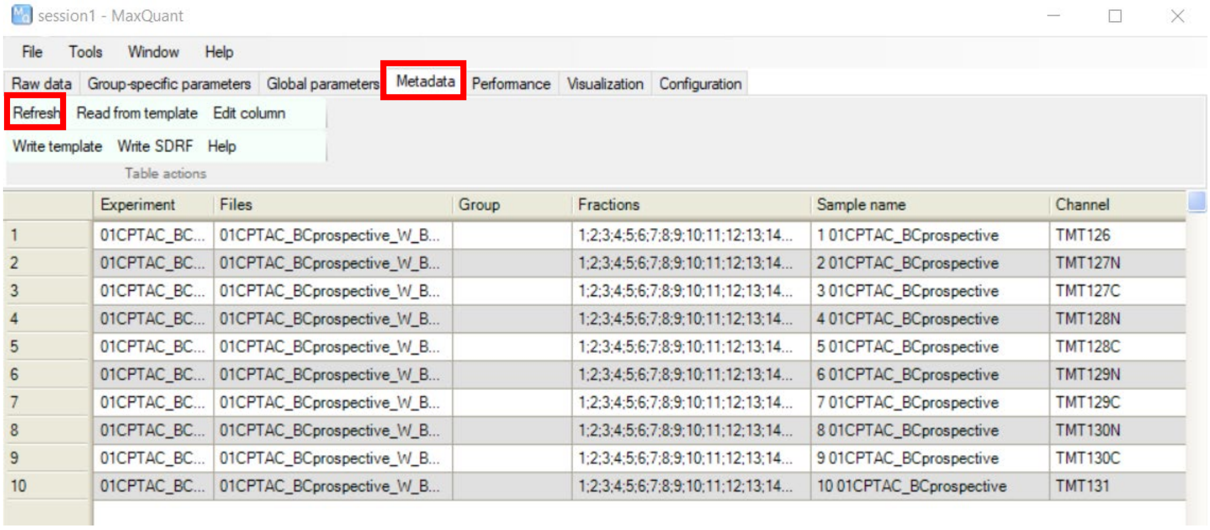

2.2 Select column(s) and edit column(s) in GUI. If information for certain columns is not available in your study, leave them empty. MaxQuant will fill the empty cells with "not available" when writing SDRF

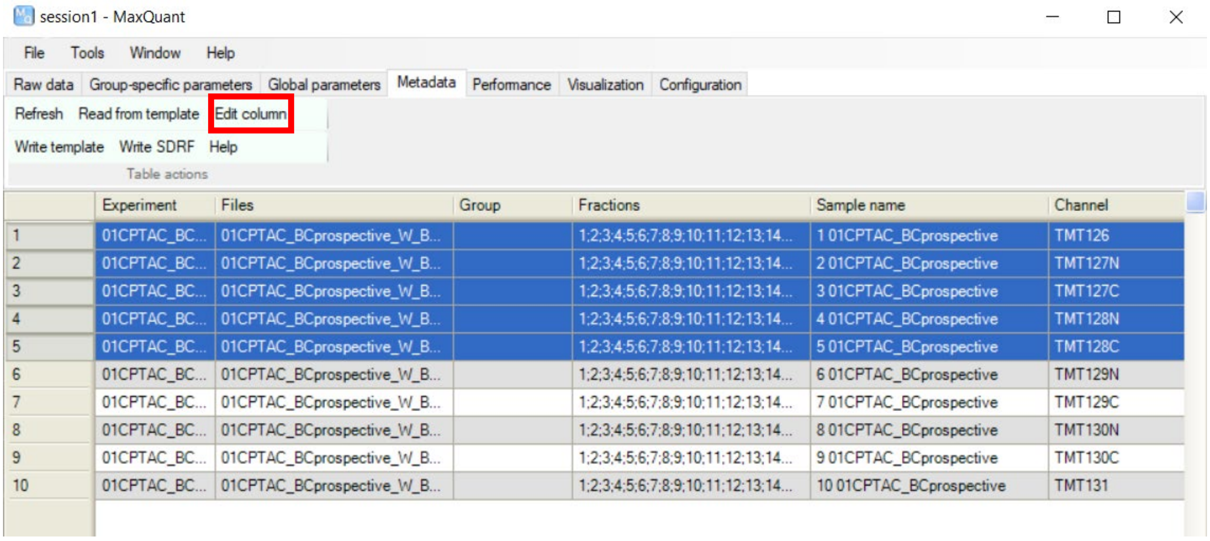

Or write template for metadata table, fill it and read from the filled template

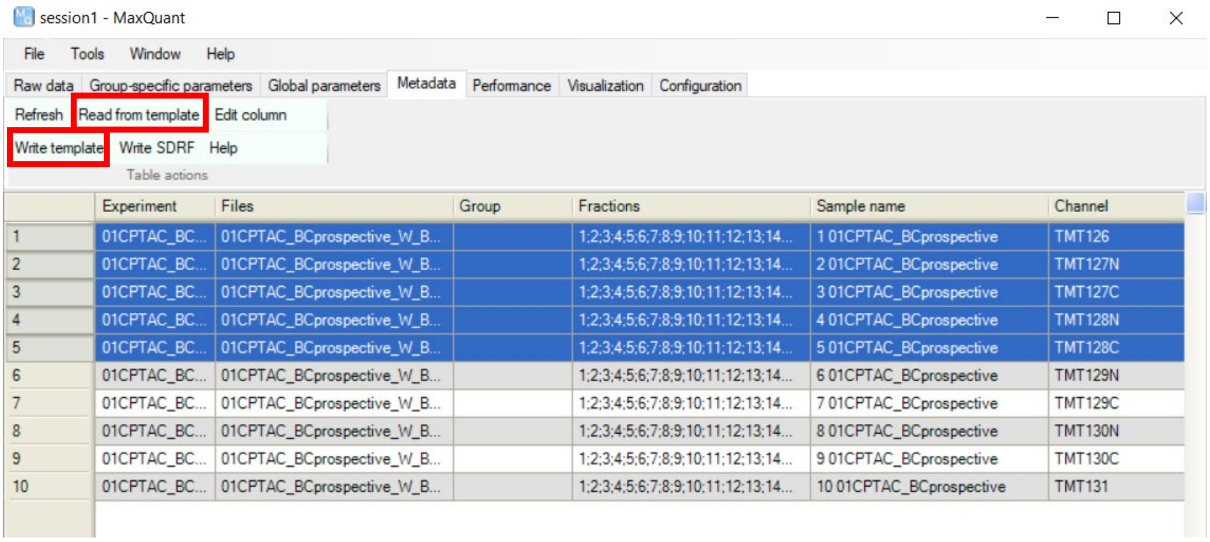

2.3 Click “Write SDRF” to write the SDRF file. The SDRF file will also be generated automatically once the MaxQuant run is complete

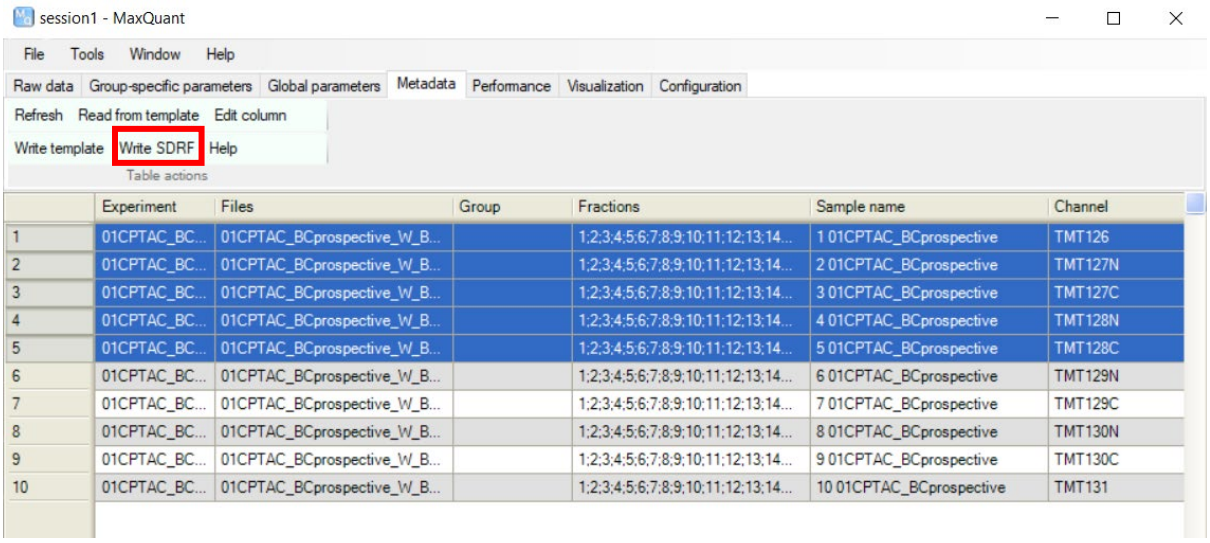

3. Annotate output tables with SDRF file in Perseus

3.1 Load the output tables in Perseus (peptides.txt, proteinGroups.txt, etc.). Specify the intensity columns as "Main"

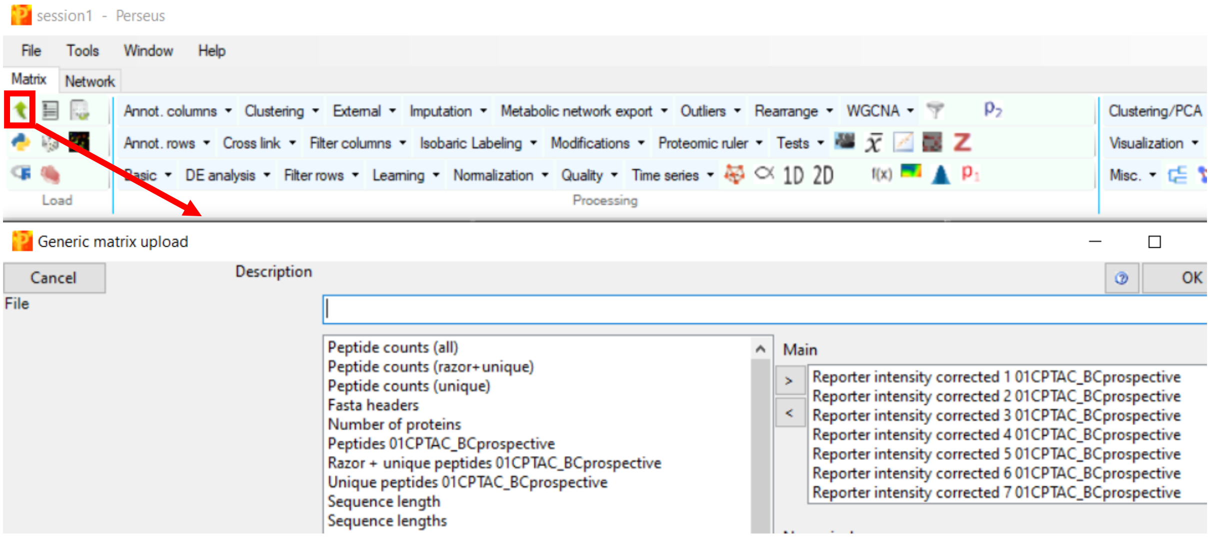

3.2 Read the SDRF file for the annotation, by default "Skip redundant properties" is selected

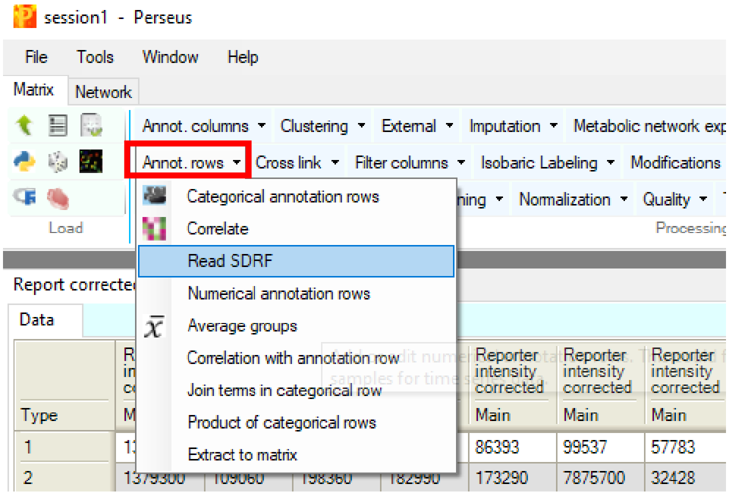

Alternatively, Python and R scripts are provided (https://github.com/cox-labs/converters) to convert MaxQuant output tables and the SDRF file into an expression matrix and a sample annotation table, which are required by other popular tools for downstream data analysis, such as DESeq2, limma, and edgeR.
