# Supplementary material for: Facilitating analysis and dissemination of proteomics data through metadata integration in MaxQuant
Source: Nat Commun. 2025 Sep 25;16:8421. doi: 10.1038/s41467-025-64089-4 (PMC12462489; doi:10.1038/s41467-025-64089-4)
Supplement: Supplementary file 2 — Description of Additional Supplementary Data [file 41467_2025_64089_MOESM2_ESM.pdf]

Supplementary Data 1

Example of SDRF file generated by MaxQuant
